# Our Insane Patients and Their Hospital Relations1Read before the Buffalo Medical Union and published by request.

**Published:** 1895-04

**Authors:** Charles A. Ring

**Affiliations:** 128 Fargo Avenue; Buffalo


### OUR INSANE PATIENTS AND THEIR HOSPITAL RELATIONS.[1]

BY CHARLES A. RING, M D., Buffalo.

ALL OF us know and fully appreciate, or have done so, the phrase "the family physician." Occupying this position of confidence and love and honor, we have felt the responsibilities and anxieties that its labors have placed upon us, and more fully have we understood as we have intrusted to the skill of some professional brother, a life held dearer than our own. The maintenance of health, through personal hygiene or municipal sanitation; the prevention of disease, either by introduction or extension, and the proper treatment of those requiring isolation, are all points that demand the attention and thought of the family physician. Further, it is his duty to know that those detailed by municipal or legislative enactment are efficient and competent in the use of means provided for special purposes; and he expects that those so employed shall furnish knowledge from their experience that shall be available to himself and to the public.

And especially are we interested in our insane patients and their hospital relations, for I think that the time has now come when the thought, opinion and wish of the general medical profession shall have more force than ever before in shaping legislation and influencing the management of the hospitals for the insane. If our patients become insane, the old question of home or hospital treatment arises: Shall we, or can we, treat them at home? May we, or must we, send them to the hospital? If at home, the case is comparatively of such infrequent occurrence that we feel the need of special authority. Where shall we look for it? If at the hospital, there may be the fears or the prejudices of the family that must be explained and removed. And, certainly, for ourselves, there are some strong objections. What assistance can we expect from those who have made the disease a special study? What objections, criticisms and suggestions can we make to those to whom we intrust our patients? What coöperation and improvement can we expect from those in charge? The duration of insanity may be so long, averaging between ten and eleven years, and its occurrences, direct, as destruction of property, assault, mutilation, homicide or suicide; and, indirect, as loss of income, loss of health of other members

1. Read before the Buffalo Medical Union and published by request.

of the family, loss of education of the children, are so serious that they demand that the patient shall be individualized, and given all that money and science can furnish.

In the annual report of the Utica State hospital for 1893, Dr. Blumer, in a table covering eleven years, shows that in those admitted who had been insane for more than one year, the percentage of recoveries was 10.53 ; of those insane less than one year, 41.22. Tables going into the months have shown that the rate of recoveries is higher, the earlier the patient has been under treatment. It is generally admitted that a case becomes chronic after two years, and that the percentage of recoveries in this class is very low, under two per cent.

Unfortunately, I do not know of statistics of recoveries of the insane in private practice. Hospital authorities are constantly urging the necessity of sending patients to them for treatment at the earliest possible moment. Yet what do we find in the following table to be the average duration of cases before commitment :

| | Under 1 month. | From 1 to 3 m'nths. | From 3 to 6 m'nths | From 6 to 9 m'nths. | From 9 to 12 m'nths. | From 12 to 18 m'nths. | From 18 to 24 m'nths. | Over 2 years. |
|---|---|---|---|---|---|---|---|---|
| | 48 | 46 | 40 | 20 | 6 | 19 | 4 | 196 |
| Utica ............ | 12.66 | 12.16 | 10.55 | 5.28 | 1.58 | 5.27 | 1.06 | 51.78 |
| | 36 | 42 | 37 | 23 | 10 | 20 | 8 | 207 |
| Willard .......... | 9.41 | 10.96 | 9.68 | 6.00 | 2.61 | 5.22 | 2.09 | 54.15 |
| | 58 | 70 | 43 | 26 | 7 | 43 | 6 | 201 |
| Hudson River ..... | 12.77 | 15.42 | 9.47 | 5.73 | 1.54 | 9.47 | 1.32 | 44.27 |
| | 54 | 31 | 34 | 26 | 8 | 33 | 9 | 148 |
| Middletown ....... | 15.71 | 9.03 | 9.91 | 7.56 | 2.30 | 9.62 | 2.62 | 43.15 |
| | 47 | 50 | 27 | 32 | 8 | 26 | 7 | 137 |
| Buffalo ........... | 14.07 | 14.97 | 8.08 | 9.58 | 2.40 | 7.78 | 2·09 | 41.02 |
| | 30 | 29 | 24 | 10 | 8 | 7 | 3 | 114 |
| Binghamton ...... | 12.77 | 12.34 | 10.21 | 4.26 | 3.40 | 2.97 | 1.27 | 52.76 |
| | 21 | 15 | 20 | 8 | 6 | 5 | 4 | 54 |
| Rochester ........ | 15.71 | 11.28 | 15.04 | 6.02 | 4.51 | 3.76 | 3.01 | 40.60 |
| | .... | .... | .... | .... | .... | .... | .... | .... |
| St. Lawrence...... | .... | .... | .... | .... | .... | .... | .... | .... |
| Number of patients. | 294 | 283 | 225 | 145 | 53 | 153 | 41 | 1,057 |
| Average per cent. of admissions | 13.30 | 12.31 | 10.42 | 6.35 | 2.62 | 6.30 | 1.92 | 46.82 |

I do not give the figures of the last annual report of the St. Lawrence state hospital, as they do not balance, and I was unable to obtain correct ones.

We find that, of 2,251 patients admitted to the state hospitals during the fiscal year 1892–93, 1,057, or 46.95 per cent., had been insane two years or over. These are practically chronic or incurable cases, as the rate of recovery among them is less than two per cent. Some of these were transferred from county asylums under the state care act, but the percentage was not large, and even these do not figure, as they had been transferred from the state to the county asylums as chronic cases. Had these 1,057 cases been placed under treatment as late as even up to one year's duration of insanity, 41.22 per cent., probably, would have recovered, but as 10.53 per cent. of all cases recover, we find 30.69 per cent., or 324 patients that might have been treated successfully, and their maintenance saved. The average cost per week of maintenance of the eight state hospitals, as reported to the State Board of Charities, was $4.56. To maintain these would cost $77,086.12 a year, and for eleven and one-half years, $886,491.40. To this direct loss of the state, must be added the indirect which a competent student of social science must estimate; and what can we say of the family circles, broken by an affliction worse than death?

Now, why is it that patients are not sooner placed in hospital care? Is it because of want of confidence on the part of the family physician, who sees and studies the various reports that are courteously sent him; or because of prejudice, or, worse yet, from fear on the part of the family that the physician is not able to overcome?

The percentage of recoveries is so low that it shows insanity to be one of the most incurable of diseases, and yet we find that the number of insane patients in the care of a single physician in the state hospitals is very much larger than in the general hospitals; and it would be larger yet, if we should take out the superintendent, who is mostly engaged in affairs of an executive character. The system of internes has been suggested, and partially adopted, but this seems to be somewhat of an evasion.

At the present time, the item for the maintenance of the insane is the largest in the yearly state budget; and up to the present time, the cost of construction per bed has increased, *and very largely*. If there had been a corresponding increase in the percentage of recoveries, the complaint might not have been so great. As long as "Gray's Folly" stands, it shall be a rebuke to the system that made the glory of the institution the point of departure, instead of the individual patient. But hold! There has been some change since the time of that most famous and successful medical lobbyist, for now, besides "The Third House," there is a "Commission" that reports directly to the legislature, and is it not rumored, or openly stated, that that "Commission" is opposed to any present appropriation for the cottage system at Collins—a system recommended by philanthropists and experts unconnected with the present system?

It would seem to me to be good financial policy for the state to appropriate enough money to secure the services of the best medical men, *and enough of them*, to meet all requirements. We can all see, physicians and legislators alike, that the interest alone on the worse than useless investments of the past would more than pay for medical attendance.

| | Cost of building. | Number of physicians. | Number of patients. | Average No. of patients to 1 physician. | Cost per bed. | Cost per week. | Per cent. of recoveries. |
|---|---|---|---|---|---|---|---|
| Utica ........ | $ 830,000.00 | 6 | 923 | 154 | $ 899.24 | $4.64 | 9.20 |
| Willard ...... | 1,134,515.00 | 9 | 2,140 | 238 | 614.25 | 2.99 | 1.90 |
| Hudson River. | 1,921,384.12 | 6 | 940 | 157 | 2,044.02 | 4.93 | 9.50 |
| Middletown .. | 1,024,500.00 | 6 | 976 | 163 | 1,049.69 | 5.57 | 10.96 |
| Buffalo ...... | 1,463,183.90 | 5 | 600 | 120 | 2,438.63 | 4.53 | 17.02 |
| Binghamton .. | 661,000.00 | 7 | 1,258 | 180 | 524.43 | 3.24 | 2.54 |
| St. Lawrence . | 1,750,000.00 | 5 | 632 | 126 | 2,768.98 | 5.20 | 11.23 |
| Rochester .... | 193,000.00 | 4 | 395 | 99 | 488.60 | 5.39 | 6.05 |

The inception and planning of Utica, Hudson River, Middletown, Buffalo and St. Lawrence State hospitals were under the old asylum system, and were carried out at an average cost of $1,839.71 per bed per patient. Willard is the result of agitation started in the New York State Medical Society in 1865, by Dr. Willard, the secretary, to remove the insane from alms-houses, and carried on by the circulation of petitions by physicians throughout the state. It was proposed to call it the Beck asylum in honor of Dr. Romeyn Beck, of Albany, author of Beck's medical jurisprudence, but Dr. Willard dying before the asylum was opened in 1869, it was named as a memorial for him. Binghamton was formerly the New York State Inebriate asylum, and Rochester, the insane department of the Monroe County almshouse. The average cost per bed per patient in these three is $542.52.

And herein comes another violation of another fundamental law of political economy, viz., that a man should do all that he can towards his own support. The insane, under an efficient system of *employment*, can do very much on the farm, in the shop and about the institution to reduce the cost of maintenance. In one year, when Willard and Binghamton were used for chronic cases, the proportion of maintenance raised by the patients was between twenty and twenty-one per cent. in *each* institution. Two or three years later, at Willard, it had fallen to sixteen. How far below the English and continental institutions ! I will not more than mention the value of systematic *employment*, not *occupation*, as one of the means of successful treatment; nor will I more than mention that class of terminal cases that an eminent German alienist termed "Recovery with defect," who, relieving the state of their support by their absence to their homes, should, in those homes, carry out the habits of industry that had been taught them in the institution. And in this connection I would say that I think there should be, on the staff of the hospital, a farmer, a practical scientific man and a graduate of an agricultural college; also a mechanical polytechnic to take charge of the shops or to start them.

As necessary as a competent medical staff is a competent corps of attendants. Much has been done to increase the efficiency of the present class of attendants in the establishment of training schools, discipline and morale. But there are essential elements wanting which cannot be obtained until better wages are paid. The attendant should be the equal of the patient socially, educationally and morally. And when this obtains, there will be few, or none, of the allegations and scandals regarding brutality towards the patient which have been so detrimental to the success of hospitals for the insane.

Of all the specialists, those connected with the insane in hospitals give us the least literature, either in the way of text-books, or papers in journals, or before medical societies. Although the *American Journal of Insanity* is one of the oldest of periodicals, its circulation is very limited among general practitioners. It has been asserted by one entirely competent, that this division of medicine has made so little progress in the last forty or fifty years, that its place in the general line of advance is practically lost, nor is its support counted upon. The cause of this is twofold. The first is that of the extreme exclusiveness and conservatism of the association of asylum superintendents, which gradually destroyed an influence that was at first all-powerful, and had the confidence and support of the public and of the profession. Second, had there been even the most liberal tendency, the medical staffs have been so small that, engaged in the general routine of the institution, there has been no time for scientific work, research and thought.

Relatives and friends visiting patients for the first time are very often unfavorably impressed by the formality and coldness of those in charge of the reception room. Sympathetic courtesy always develops thoughts and feelings which approve and sustain discipline. On this first visit they expect to see the one called for within a few minutes, and as the time passes to half an hour, then to an hour or longer, impatience passes into suspicion and anxiety, and by the time the patient arrives, they are often ready to believe that he has been and is kept in an unpresentable state. And if the patient makes numerous complaints, or gives his opinions as to the daily life and occurrences of the ward, they are all accepted as confirming the suspicions.

Too frequently there are deaths in hospitals for the insane under very suspicious circumstances. The post mortem examinations show contusions, fractures and other injuries that could hardly have been produced by the patient himself. The press reports the matter as of the daily news or occurrences which are more or less interesting to the public. And people ask, "Why do not the authorities know what is going on? Why are not the guilty detected? Why is it that we never hear of legal punishment?" And the people are right in asking these questions. There are peculiar secret means that can be used to obtain corroborative testimony; and, if necessary, competent testimony from patients can be introduced. And especial legislative enactments have been made for the punishment of those offending against the insane. The detection of offenses by the hospital authorities and the furnishing of evidence for prosecution and conviction would be assurance which the public would most gladly accept. The trial might show peculiar revelations, but the press could be depended upon to present the matter fairly to the public for its hought and judgment.

I think it is a great mistake that the press is not taken into the full confidence of the institution, and its corresponding coöperation or protection secured.

128 Fargo Avenue.

